# Supplementary material for: Complexome profiling of the Chlamydomonas psb28 mutant reveals TEF5 as an early PSII assembly factor
Source: Plant Cell. 2025 Mar 17;37(6):koaf055. doi: 10.1093/plcell/koaf055 (PMC12164590; doi:10.1093/plcell/koaf055)
Supplement: koaf055_Supplementary_Data [file koaf055_supplementary_data.zip › psb28_interactive.zip!/plots/intensity/qualityPlots/peptides/DHC1_Cre12.g484250.t1.1.html]

### Cre12.g484250.t1.1

Trivial name: DHC1  
SeqCoverage [median]: 0.60  
Mass: 522.88 kDa  
  
 

| Sequence | MissedCleavage | Mass [Da] | wtIntensity | muIntensity | Score |
| --- | --- | --- | --- | --- | --- |
| ELDAVTESQLR | 0 | 1259.64 | 93617.77 | 160648.36 | 51.93 |
| ELTIESEIRKLADVWR | 2 | 1957.06 | 3670.14 | 12835.39 | 28.09 |
|  |  |  | 97287.9 | 173483.8 |  |
